# Expression of a Gene Encoding 34.9 kDa PPE Antigen of *Mycobacterium avium* subsp. *paratuberculosis* in *E. coli*

**DOI:** 10.4061/2010/628153

**Published:** 2010-05-18

**Authors:** Rajib Deb, P. P. Goswami

**Affiliations:** Gene Expression Laboratory, Division of Animal Biotechnology, Indian Veterinary Research Institute, Izatnagar, Uttar Pradesh 243 122, India

## Abstract

*Mycobacterium avium* subsp. *paratuberculosis* (Map) contains PPE family antigens which are Proline and glutamic acid rich and may play important role as T cell antigens. Hence the identification and generation of antigens are necessary for immunological characterization. In the present study, the epitopic region of a unique PPE gene encoding 34.9 kDa protein from Map was amplified by polymerase chain reaction. The gene was cloned into *Escherichia coli* vector pQE30 UA. The recombinant plasmid designated as pQPPE was transformed into *E. coli* M15 and induced with IPTG revealed the high level expression of 37.1 kDa His-fusion protein (34.9 kDa PPE and 2.2 kDa His-tag), which was confirmed by immunoblotting. Recombinant PPE protein was then purified by Ni-NTA agarose chromatography. The polyclonal antiserum raised against purified recombinant PPE protein reacted with expressed 37.1 kDa His-fusion protein as well as with Map sonicate. The protein elicited significant delayed type hypersensitivity (DTH) skin reaction in mice sensitized with Map. The results indicated that the recombinant PPE protein of Map was associated with cellular immune response.

## 1. Introduction

*Mycobacterium avium *subsp.* paratuberculosis *(Map) is the main causative agent of paratuberculosis, which is a chronic disease of ruminants [1]. Although disease associated with Map infection is largely restricted to ruminant species, the organism has also been implicated as a causal or exacerbating agent in human Crohn's disease [2–7]. The search for Map specific antigens for diagnostic or preventive therapy has led to the discovery of several immunoreactive proteins. Many of these proteins have homology to other mycobacterial antigens like 44.3 kDa soluble protein [8], GRO ES a heat shock protein [9], Alkyl hydroperoxide reductase D [10], 85B [11], SOD [12], 35 kDa [13, 14], 16.8 kDa [15, 16], hsp70 [17], Mycolyl Transferase [18], Pro-Glu rich PE proteins [19], and some PPE family proteins [20–23].

The sequencing of *Mycobacterium tuberculosis *(Mtb) revealed two large families of PE and PPE proteins, which are Pro-Glu rich and constitute approximately 10% of the coding capacity of the genome [24]. However, no precise function is known for any members of these families, some members in Mtb have been found to associate with the cell wall and to influence interaction with other cells [25]. It has also been suggested that members of PPE proteins play a role in immune invasion and antigen variation or may be linked to virulence and some have been found associated with the binding to eukaryotic cells, survival within macrophages and persistence in granulomas [22, 26, 27]. Okkels et al. reported that the few proteins encoded by the Mtb Rv3873, Mtb39, and Mtb Rv0915c genes have been shown to be T cell antigens [28]. Later Chaitra et al. [29] demonstrated that the recombinant proteins encoded by the MtbRv 3108c and Rv 3812 genes of the PE/PPE families are the T cell antigens. Thus the PPE protein family has gained much interest as a promising target for molecular characterization [29].

There are at least thirty seven PPE proteins coding sequences present in the Map genome [30], but no information on their putative role exists. All of the PPE protein family of Map have a conserved N-terminal domain that consists of 180 to 200 amino acid residues. Recently some of the PPE proteins of Map encoded by Map 41 and Map 39 genes have been reported in inducing IFN*γ* [31]. Hence the identification of specific PPE antigen that stimulates the T cell response from PPE protein family of Map seems to be an initial requirement for the development of diagnostic tests based on cell mediated immune (CMI) response and vaccine. In the present study, we describe the heterologous expression, purification, and preliminary characterization of epitopic region of 34.9 kDa PPE protein of Map.

## 2. Materials and Methods

### 2.1. Bacterial Strains and Plasmid

*M. a. paratuberculosis *strain 316F was obtained from Central Diengenees Kundig Tieh Institute, Lelystad, The Netherlands, and maintained at Gene Expression Laboratory, Animal Biotechnology Division, IVRI, Izatnagar, India. The mycobacteria were grown and maintained at 37°C on Middlebrook 7H10 agar (Difco laboratories, Detroit, USA) enriched with 0.1% glycerol (v/v) and 10% oleic acid dextrose catalase (Difco laboratoriesand supplemented with of 20 mg/L mycobactin J (Allied Monitor, Fayette, USA) was also included. *E. coli* strain (M15, pREP4) supplied by Qiagen (Valencia, USA) was grown at 37°C in Luria Bertani broth containing kanamycin (25 *μ*g/mL), as the strain carries kanamycin resistant plasmid. The expression vector plasmid pQE30 UA was purchased from Qiagen. The plasmid contains a T5 promoter and a 6 × His-tag coding sequence at 5′ to the multiple cloning region. The plasmid also contains an ampicillin resistance marker.

 To amplify a 34.9 kDa PPE protein of Map, the primers: pIRES Map PPE F (sense) 5′-GCC GCT AGC ATG TGG GTC CAG GCC GCC AC-3′-29 mer and pIRES Map PPE R (antisense) 5′-GCC GAA TTC TTA CTC GGT TCC AGC GTT GC-3′-29 mer, respectively, containing *Nhe*I and *EcoR*I restriction endonuclease sites were designed on the basis of sequence information of Map str. k10, complete genome Gene Bank Accession No. AE016958 tag Map 3737 region 4159938–4160683.

### 2.2. Preparation of DNA

The genomic DNA from *M. a. paratuberculosis* was isolated from the grown culture by the method of Portillo et al. [32]. Plasmid DNA extraction from *E. coli* was carried out by the alkaline lysis method [33].

### 2.3. PCR Amplification and Cloning

The amplification reaction was performed in a 25 *μ*L reaction volume containing 100 ng of *M. a. paratuberculosis* DNA; 2.5 *μ*L of *Taq* DNA polymerase buffer 10 mM/L Tris.HCl (pH 9.0), 50 mM/L KCl, 1.5 mM/L MgCl_2_ and 0.01% (w/v) gelatin; 200 *μ*M of each dNTP; 0.5 *μ*M each primers; 1 units of *Taq* DNA polymerase. The final volume was made up with sterile distilled water. 

 The reaction was carried out in a PTC-100 reactor (MJ Research Inc., Waltham, USA) for 30 cycles each cycle consisting of denaturation at 94°C for 1 minute, annealing at 55°C for 1 minute, and extension at 72°C for 1 minute. The amplified product was analyzed by submarine gel electrophoresis (Genei, Bangalore, India) on 1% agarose gel.

The amplified gene product was purified from agarose gel using a QIAEXII gel extraction kit (Qiagen). For the purpose of cloning, about 500 ng of the PCR product was ligated into the plasmid pQE30UA expression vector. The resulting plasmid pQPPE was transformed into competent* E. coli* M15 cells. The recombinant clones were selected on LB agar containing ampicillin (75 *μ*g/mL) and kanamycin (25 *μ*g/mL). Transformants were further screened by restriction enzyme analysis of the plasmids with *Nhe*I and *EcoR*I [33]. Lambda EcoRI/Hind III digest and lambda ladder 100 bp ladder were used as standard molecular weight marker for agarose gel electrophoresis (Genei).

### 2.4. Expression and Purification of the Recombinant 37.1 kDa His-Fusion Protein

*E. coli *M 15 cells harbouring the plasmid pQPPE were grown in LB medium containing (75 *μ*g/mL) and kanamycin (25 *μ*g/mL) and induced with 1.0 mM IPTG for 4–6 hours. The purification of recombinant 37.1 kDa (34.9 kDa PPE and 2.2 kDa His-tag) His-fusion protein under denaturing conditions was carried out by single step affinity chromatography using Ni-NTA (nickel-nitrilotriacetate) agarose (Qiagen) and renatured as described previously [13]. The protein concentration was determined spectrophotometrically [34].The protein solution was sterilized by filtration, and aliquots were stored at −70°C, until used.

### 2.5. Antibody Production

Two New Zealand White rabbits (8–10 weeks old) obtained from Laboratory Animals Resource section, IVRI, Izatnagar were used to raise antibody against the recombinant 37.1 kDa His-fusion protein. Rabbits were immunized subcutaneously with 150 *μ*g of immunogen with incomplete Freund's adjuvant (IFA) (Genei, Bangalore, India), and boosters of 100 *μ*g of the immunogens with IFA were given intramuscularly after 3 weeks and again 2 weeks later. Animals were bled ten days after the second booster and sera samples separated and stored at −20°C. All the sera used in this study were preabsorbed with* E. coli* antigens following the procedure of Harlow and Lane [35]. 

 In brief, recombinant 37.1 kDa His-fusion protein was initially electrophoresed and transferred to nitrocellulose membrane. The specific protein band was excised from the blot and incubated with polyclonal rabbit serum diluted 1 : 3 in buffer I (0.1 M NaH_2_PO_4_, 0.01 M Tris.Cl, pH 7.5) at 37°C, overnight. Following washing the membrane in buffer I, the monospecific antibodies were eluted in buffer II (0.1 M NaH_2_PO_4_, 0.01 M Tris.Cl, pH 6.3). These were utilized in western blotting experiments.

### 2.6. SDS-PAGE and Western Blotting

SDS-PAGE of the expressed recombinant 37.1 kDa His-fusion protein was carried out on a vertical slab miniapparatus (Atto, Tokyo, Japan) using polyacrylamide gels run under denaturing conditions as described by Laemmli in [36]. The 12% separating and 4% stacking polyacrylamide gel contained 0.1% SDS. About 25 *μ*g of each sample was boiled in equal volume of 2X sample loading buffer prior to loading. Electrophoresis was carried with Tris-glycine electrode buffer (1X), pH 8.3 at 90V for 2 hours; the gels were stained overnight with Coomassie brilliant blue G 250 (Sigma, Milwaukee, USA) [37]. The protein marker range from 20 to 120 kDa (MBI, Fermentas, Germany) was used as size markers.

Samples electrophoresed on 12% SDS-PAGE were transferred to nitrocellulose membranes (0.45 *μ*M) using semidry electroblotting (Atto, Tokyo, Japan) at 0.8 mA/cm^2^, following the method of Bjerrum and Schaffer-Nielsen [38]. The blots were blocked with 2% skimmed milk powder in PBS-T buffer (PBS containing 0.1% Tween-20) for 2 hours at room temperature. After washing with PBS-T buffer three times, the membranes were incubated for 2 hours at 37°C with antisera aganist 37.1 kDa His-fusion protein (1 : 000 in PBS) raised in rabbit. Following further washing, the blots were incubated with a 1 : 1000 dilution of HRP-labelled goat antirabbit IgG (Genei) for 1 hour. After washing, the blot was dipped in substrate solution (0.02% diamino benzidine suspended in PBS containing 0.03% hydrogen peroxide in PBS pH 7.4) for a minute, till brown colour developed.

### 2.7. Dot Blot Assay

About 25 *μ*g of recombinant 37.1 kDa His-fusion protein in 25 *μ*L PBS and Map culture sonicate were put on nitrocellulose membrane dried and allowed to react with polyclonal antisera against recombinant 37.1 kDa His-fusion protein as per the method described in western blot. 

### 2.8. Nucleotide Sequencing and Deduced Amino Acid Analysis

The nucleotide sequence determined of the 1080 bp gene encoding PPE protein of Map strain 316F has been deposited in nucleotide database. The deduced amino acid sequence of gene encoding 34.9 kDa PPE protein from Map was analyzed for hydrophobic domains according to Kyte and Doolittle algorithm [39] using Lasergene software (DNASTAR, Madison, USA). The homology prediction was also done with the help of public server UNIPROT and PSI-BLAST.

### 2.9. Measurement of Delayed Type Hypersensitivity

Pathogen free twenty four female Swiss albino mice (6–8 weeks old) divided into two groups consisting of twelve animals each. Group I was subcutaneously injected with heat killed *M. a. paratuberculosis* 316F (100 *μ*g/animal in PBS) mixed with sterile incomplete Freund's adjuvant (IFA) (1 : 1), while group II (control) was immunized with PBS-IFA alone. Two weeks later the mice were again immunized in the same way. After 4 weeks of the first injection all the mice were injected intradermally with 10 *μ*g of purified recombinant 37.1 kDa His-fusion protein in (10 *μ*L PBS) in right hind foot pad and 10 *μ*g johnin PPD in the left hind foot pad. Four mice each from both groups were also injected with 10 *μ*L PBS as negative control. The results of the local skin reactions (DTH) were read after 48 hours by measuring the two transverse diameters of erythema and swelling; the mean (±SEM) of which is quoted in the results. Differences between treatment means were assessed for significance by Student's *t*test, at a significance level of *P* < .05.

## 3. Results

### 3.1. PCR Amplification and Construction of Recombinant pQPPE

**Figure 1:**
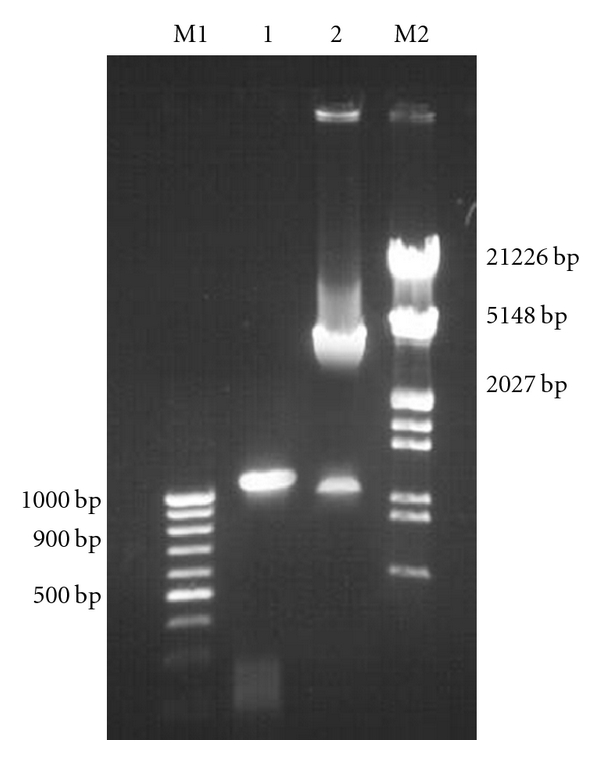
Agarose gel 1% of the clone fragment of the gene PPE (1080 bp) in pQE-30 UA expression vector. Lane M1: DNA molecular weight marker 100 bp ladder. Lane1: PCR amplified PPE gene fragment of *Mycobacterium avium *subsp*. paratuberculosis.* Lane 2: Released insert of 1080 bp PPE gene fragment after digestion with NheI and EcoRI from recombinant plasmid pQE-30 UA PPE. Lane M1: Presented DNA molecular weight marker Lamda DNA/EcoRI/Hind III digest.

A PCR product of 1098 bp (1080 bp PPE gene and 18 bp linker) was obtained on amplification at 55°C. Restriction digestion of the recombinant plasmid pQPPE with NheI and EcoRI released an identical size fragment as seen on a 1% agarose gel (Figure 1).

### 3.2. Expression of Recombinant 37.1 kDa His-Fusion Protein in *E. coli*

**Figure 2:**
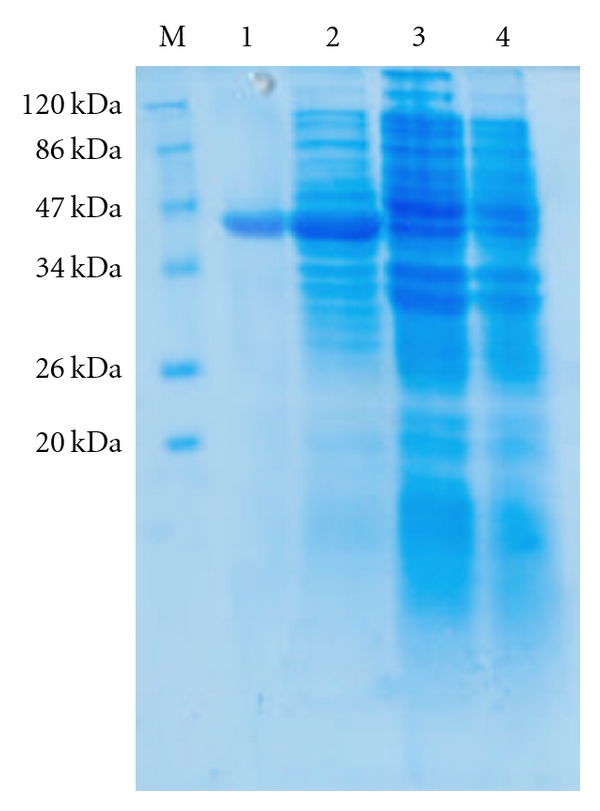
Coomassie Brilliant Blue stained 15% SDS-PAGE showing expressed PPE protein. Lane M: Prestained protein molecular weight marker. Lane 1: Purified recombinant 37.1 kDa his tag recombinant PPE protein. Lane 2: Whole cell extract of *E. coli *M15 harbouring 37.1 kDa fusion PPE-6xhistag protein (IPTG induced). Lane 3: Whole cell extract of *E. coli *M15 harbouring 37.1 kDa fusion PPE-6xhistag protein (IPTG uninduced). Lane 4: Whole cell extract of *E. coli *M15.

When *E. coli* M15 cells harbouring the recombinant plasmid pQPPE were induced for 6 hours with 1 mM/L IPTG and analysed by 12% SDS-PAGE and Coomassie staining, a predominant band corresponding to that predicted for 37.1 kDa His-fusion protein was detected in total cell extract of the *E. coli* (Figure 2, lane 2). The recombinant protein formed a major (more than 30%) portion of the total *E. coli* extract. No such protein band was observed with *E. coli* M15 cells or in uninduced *E. coli* M15 cells harbouring recombinant plasmid pQPPE (Figure 2, Lanes 3 and 4).

### 3.3. Purification of the Recombinant 37.1 kDa His-Fusion Protein

The His-tagged purified protein, when analyzed on SDS-PAGE, showed a monomeric band of about 37.1 kDa size (Figure 2, Lane 1). Purification of the recombinant protein was nearly 80%, as visualized on SDS-PAGE. The yield of the pure recombinant protein was about 15–20 mg/L of culture at shake flask level.

### 3.4. Immunoreactivity of the Recombinant Protein

**Figure 3:**
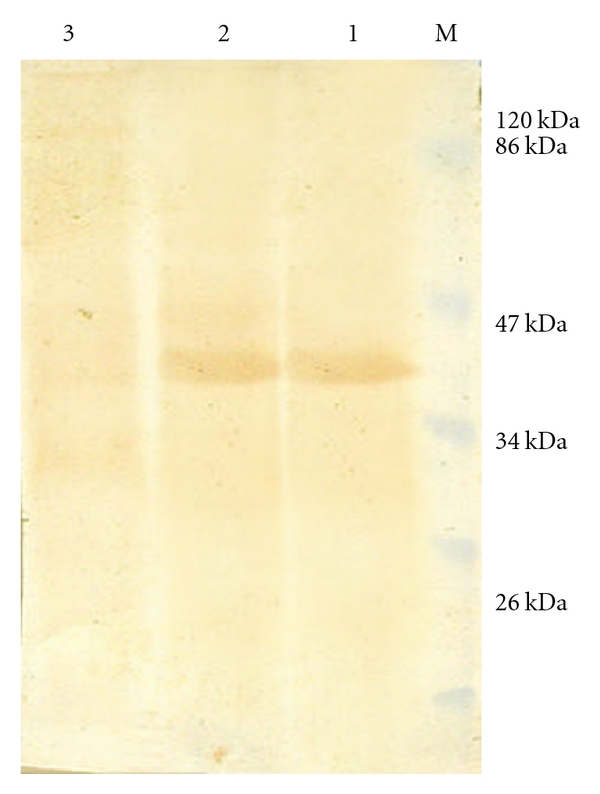
Western blot assay of the PPE protein expressed in *E. coli *cells. Lane M: Prestained protein molecular weight marker. Lane 1: Purified recombinant PPE-protein (37.1 kDa). Lane 2: Whole cell extract of *E. coli* M15 harbouring 37.1 kDa fusion PPE-6xhistag protein (IPTG induced). Lane 3: Whole cell extract of *E. coli* M15 harbouring 37.1 kDa fusion PPE-6xhistag protein (IPTG uninduced).

**Figure 4:**
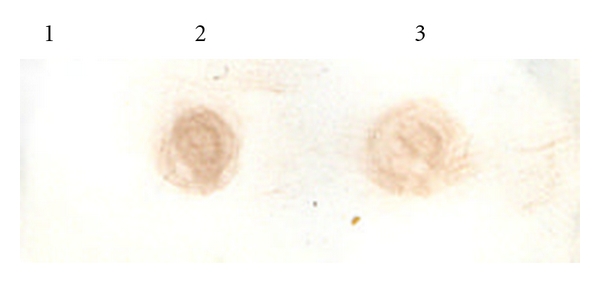
Dot blot assay showing sero reactivity of polyclonal sera against PPE 37.1 kDa recombinant protein with purified recombinant PPE protein and *Mycobacterium avium *subsp*. paratuberculosisi * strain 316F sonicate. Lane 1: PBS negative control. Lane 2: Recombinant purified 37.1 kDa PPE. Lane 3: *M. a. paratuberculosis *strain 316F sonicate.

The polyclonal antiserum raised against purified 37.1 kDa His-fusion protein in rabbits could bind to the IPTG that induced whole cell extract expressing recombinant as well as purified 37.1 kDa His-fusion protein on western blot (Figure 3, Lane**s** 2 and 1). However no such band was visible in uninduced total cell extract of M15 harbouring pQPPE plasmid (Figure 3, Lane 3). Furthermore, the serum could also bind to *M. a. paratuberculosis *sonicate on dot blot (Figure 4, Lane 3).

### 3.5. Deduced Amino Acid Analysis

The nucleotide sequence of the pQ PPE plasmid having 1080 bp PPE gene of Map has been deposited in Gene bank database under accession no. FJ032182. The Blast search of the sequence revealed that no other mycobacterial sequences have homology. The predicted 359 amino acids of the 1080 bp gene fragment had a mature protein of 34.9 kDa. Analysis of the deduced 359 amino acids sequence indicated that the protein contained three major hydrophobic regions (amino acids 25–30, 245–255, and 335–350).

### 3.6. Induction of DTH Response

Mice sensitized with *M. a. paratuberculosis *(316F) reacted with 37.1 kDa recombinant PPE protein giving skin reaction. However, response to johnin PPD was greater than that of recombinant 37.1 kDa His-fusion protein, unsensitized animals did not show significant skin reactions to 37.1 kDa His-fusion protein as well as johnin PPD (Table 1).

## 4. Discussion

Understanding the immune response elicited by the PPE protein family of Map requires the identification of the antigen(s) or epitopes. A few antigens from the PPE family of Mtb and Map have been reported to elicit T cell response [31, 40, 41] or some of them are involved to mediate humoral immune response [22, 31]. However, the protein encoded by the Mtb Rv 3425 gene has been shown to induce both humoral and cellular immune response [42]. The completion of Map genome has accelerated dramatically in the biology of this significant pathogen and also provided information about several ORFs from PPE family proteins. In the present study, we have selected a unique sequence of 1080 bp from the Map genome with no homology to other mycobacteria and efficient expression system, based on the pQE30 UA vector, to produce 34.9 kDa protein of Map in *E. coli,* so as to facilitate further characterization of the protein.

Based on the sequence information of the gene encoding 34.9 kDa protein of Map str. k10, complete genome Gene Bank Accession No. AE016958 (tag Map 3737 region 4159938–4160683 coding for hypothetical PPE protein) and also the information on the multiple cloning site of the expression vector pQE30 UA vector, restriction sites for *Nhe*I and *EcoR*I were incorporated into the primers to facilitate directional cloning. In the design of the forward primer, consideration was given to inserting the amplified gene of 34.9 kDa protein in frame into the pQE30 UA vector under the T5 promoter. The resulting plasmid pQPPE contained an open reading frame encoding successively 6X His polypeptide and the 34.9 kDa protein. The recombinant PQPPE clones were confirmed by release of the insert by double digestion with *Nhe*I and *EcoR*I enzymes and on induction with IPTG appearance of the 37.1 kDa band consistent with the predicted size of 34.9 kDa protein.

A similar strategy of cloning in pQE series expression vector system was applied to generate high levels of 35 kDa protein of Map [13] and a 26 kDa protein of *Brucella abortus *[43]. Expression of recombinant proteins is induced by, IPTG which binds to the *lac* repressor protein, inactivating it leading to transcription of sequences downstream of the promoter. 

Expression of the gene facilitated the production of large amounts of the recombinant protein for immunological studies. The presence of 6X-histidine tag is of 2.9 kDa size at N-terminal of the recombinant protein facilitated single step affinity purification. It rarely interferes with protein immunogenicity, protein functional structure, hence the tag was not removed by protease cleavage [44].

 The polyclonal sera raised in rabbit against the recombinant His-PPE 37.1 kDa protein recognized the *E. coli* expressed recombinant PPE protein and sonicate fractions of Map on immunoblot. This had not only confirmed the heterologous expression of the 34.9 kDa Map PPE protein but also suggested that recombinant fusion protein retained its Antigenicity.

The analysis of the deduced amino acid sequence according to the Kyte and Doolittle algorithm [40], showed the presence of three highly hydrophobic regions, which could be the membrane segment of the protein. The homology study and deduced amino acid sequence are unique to Map and have the potential to be developed in diagnostic reagent.

The DTH responses were observed in all the animals sensitized with Map using recombinant PPE protein as well as johnin PPD. The greater responses to Johnin PPD may be due to polyclonal activation of T cells by multiple antigenic components in these preparations, compared to the single antigenic component of the recombinant PPE protein. The role of recombinant PPE protein in eliciting DTH response in naturally infected animals needs to be investigated.

Further studies are in progress to investigate the in vitro CMI response to the recombinant PPE protein with the aim of evaluating its use in cellular immunity-based diagnostic test and/or immunoprophylactic for paratuberculosis.

## Figures and Tables

**Table 1:** DTH elicited by purified recombinant PPE protein in *M. a. paratuberculosis* sensitized mice (mean diameter of erythema in mm) upon recall with 10 *μ*g of the indicated antigen.

	Antigen		
Sensitizing organism	Johnin PPD	Recombinant PPE protein	PBS
*M. a. paratuberculosis*	3.95 ± 0.5	3.12 ± 0.4	0.18 ± 0.2
Unsensitized	0.17 ± 0.3	0.13 ± 0.2	0.13 ± 0.5
